# Flow in choral singing: associations with perceived choral memory performance and well-being among older adults

**DOI:** 10.1186/s40359-025-03276-w

**Published:** 2025-08-11

**Authors:** Pengcheng Su, Kuaian Jiang, Jiayin Kong

**Affiliations:** 1 Guangdong University of Petrochemical Technology, No. 1 Kechuang Road, Maoming, Guangdong Province China; 2 College of Music, Mahidol University, 25/25 Phutthamonthon Sai 4 Rd, Salaya, Phutthamonthon District, Nakhon Pathom, 73170 Thailand; 3 Pathumthani University, 140 Santi Suk Mu 4 Alley, Ban Klang, Mueang Pathum Thani District, Pathum Thani, 12000 Thailand

**Keywords:** Flow, Older adults, Choral environment, Perceived choral memory performance, Well-being, SEM

## Abstract

Drawing on flow theory, this study examines relationships between choral environment, song style, and flow, proposing a choral experience model to investigate associations with perceived choral memory performance and well-being among older choir members. We employed structural equation modeling (SEM) to analyze data from 412 older adult participants in amateur choral groups across China. Results reveal significant positive associations between five factors (skill-challenge balance, clear goals, immediate feedback, song style, and choral environment) and flow. Notably, the choral environment demonstrated the strongest association with flow. Furthermore, our analysis identified both direct relationships between flow and well-being, and an indirect relationship through perceived choral memory performance. The study also revealed gender differences, with females reporting higher well-being scores than males. By examining flow within the choral context of aging populations, this research extends theoretical understanding of how collective musical engagement relates to cognitive and emotional outcomes. These findings suggest valuable considerations for choir directors, health professionals, and policymakers interested in the potential benefits of supportive choral environments and participation for older adult well-being and perceived choral memory performance.

**Supplementary Information:**

The online version contains supplementary material available at 10.1186/s40359-025-03276-w.

## Introduction

Extensive research in music psychology highlights the significant association between musical activities and both psychological and physical health. Among these, choral singing stands out for its combination of social interaction, music listening, and collective performance, offering a holistic approach connected to enhanced well-being [1, 2]. Despite its well-documented benefits, the exploration of ‘flow’ experiences–a state of complete immersion and focused engagement [3]--in choral settings, particularly among older adults, remains underexplored. This gap is surprising, given the potential of choral singing to relate to deep psychological engagement and emotional fulfillment in older adults. This stands in contrast to younger musicians or solo performers, whose flow experiences often correlate with individual skill mastery [4, 5].

Previous research has largely examined related areas, including musical performance anxiety [6], live performances [7], composition processes [8, 9], group creativity [10], and the interplay between music and sports [11, 12]. While these studies have advanced our understanding of ‘flow’ in various contexts, they have largely overlooked the unique experiences of older adults in choral settings [13]. This oversight is particularly significant, as older adults may exhibit distinctive patterns in choral singing corresponding to their specific psychological and social needs, such as cognitive decline, physical limitations, and social isolation. Moreover, cultural factors, like the collectivist resonance of Chinese ‘red songs,’ may uniquely relate to flow in this population, suggesting a need to extend traditional models [3].

Empirical studies consistently highlight the correlates of ‘flow,’ including positive emotions, greater satisfaction [5, 12, 14, 15], and subjective well-being [1, 16]. ‘Flow’ has also been associated with heightened concentration [11, 13, 17], memory retention [17], and intrinsic motivation [18]. However, these findings often fail to account for the unique contextual factors related to older adults, raising questions about the generalizability of existing research to this population. In the Chinese choral context, for instance, cultural elements may correspond to different patterns of flow, offering a fresh perspective on its dynamics.

This study addresses three key research questions:

1. *What factors are associated with ‘flow’ experiences among older adults in choral singing*,* particularly the relationships between skill-challenge balance*,* clear goals*,* immediate feedback*,* song style*,* choral environment*,* and flow?*
2. *How do ‘flow’ experiences relate to perceived choral memory performance and well-being in this population?*
3. *How does gender correspond to different patterns in the relationship between choral participation and well-being in this population?*

By addressing these questions, this study aims to provide novel insights into the patterns connecting ‘flow’ with psychological and social well-being of older adults, potentially extending flow theory by integrating socio-cultural dimensions specific to Chinese choral settings. The findings aim to provide practical guidance for choral organizers, music therapists, and older adults care providers. These insights can inform the design of choir environments, selection of suitable song styles, and approaches to promoting cognitive and emotional health through choral singing.

## Literature review

### Flow

The concept of ‘flow,’ introduced by Csíkszentmihályi [3], describes a state of complete immersion and focused engagement. This experience is characterized by clear goals, immediate feedback, and a balance between skill and challenge [13]. These factors are associated with a heightened state of consciousness, allowing individuals to lose track of time and derive intrinsic enjoyment from their activities.

In the field of music psychology, ‘flow’ has garnered significant attention [11]. Studies have identified elements such as skill-challenge balance, autonomy, and intrinsic motivation as factors associated with flow in music performance [5, 8, 19]. However, much of this research has been conducted with individual performers, overlooking group contexts like choral singing, where social dynamics play a pivotal role [13, 20]. Empirical studies on group flow emphasize the importance of shared goals, effective communication, and emotional contagion in relation to collective flow [20]. Yet, these findings have rarely been applied to older adult populations in choral environments, raising concerns about their generalizability to this demographic.

In music education, ‘flow’ has been observed alongside enhanced motivation, skill development, and self-efficacy among students [5, 18, 21]. However, the existing literature predominantly focuses on younger, musically trained individuals, leaving a significant gap in understanding how older adults, particularly those participating in choral singing, experience flow. This gap is critical because older adults, as a diverse group, may face a range of experiences, including potential age-related cognitive decline and physical limitations, which may relate to different patterns of flow [17].

In summary, while research on ‘flow’ has deepened our understanding of its correlates and associated outcomes in music, the manifestation of flow among older adults in choral settings remains underexplored. Addressing this gap could offer valuable insights for developing approaches aimed at enhancing well-being and cognitive health through musical engagement.

### Theoretical model and research hypotheses development

#### Factors associated with flow (Skill-challenge balance, clear goals, immediate Feedback)

This study investigates factors associated with flow among older adults in choral settings and examines how flow relates to perceived choral memory performance and overall well-being. To do so, we ground our model in Csíkszentmihályi’s flow theory [3],

making a critical distinction between the conditions (or antecedents) that facilitate entry into a flow state, and the subjective experiential dimensions that characterize the state itself. Specifically, we examine three core conditions well-established in the theory: skill-challenge balance, clear goals, and immediate feedback. The flow experience itself is then captured through its core experiential dimensions (e.g., deep concentration, intrinsic enjoyment). This framework allows us to empirically test a core tenet of flow theory: whether the established theoretical conditions for flow are indeed associated with the experience of flow.

While these constructs are well-established, their application to older adults in choral contexts remains underexplored. Furthermore, it is important to note that given the cross-sectional nature of this study, we explore the correlational patterns between these factors and flow, rather than strict causal relationships. This approach better aligns with the empirical limitations of the present research while still providing valuable insights into flow in older adult choral contexts.

Skill-challenge balance is widely recognized as being associated with flow [2, 4, 5, 22]. However, its operationalization for older adults presents unique challenges. As Agres and colleagues [17] caution, age-related cognitive decline may correspond to different patterns in maintaining this balance, potentially relating to increased susceptibility to anxiety or boredom. Approaches must therefore be carefully designed to ensure that tasks are neither too difficult nor too easy, addressing these potential barriers to flow. Although the relationship between skill-challenge balance and flow is well-documented, its practical implementation in aging populations requires further empirical investigation.

Clear goals are another essential component related to flow, corresponding to direction and motivation. In group settings such as choral singing, however, goal-setting can be complex. Ding and Hung [4] emphasize that clear goals are associated with focus and motivation, while Peifer and colleagues [21] note that overly rigid goals may correlate with suppressed creativity, particularly in collaborative environments. Daffern and colleagues [23] further argue that goal clarity should be balanced with flexibility to accommodate the diverse skill levels and preferences of older adults. This tension between structure and adaptability highlights a gap in current research, which often assumes uniformity in goal-setting processes. For older adults, inclusive and adaptable goal-setting strategies may be associated with flow in choral contexts.

Immediate feedback is observed alongside sustained flow, as it correlates with enhanced self-efficacy, performance, and clear indicators of progress [2, 5, 18, 21]. However, in group settings, the relationship between feedback and flow depends on the quality and source of feedback. In choral environments, feedback operates on multiple levels: from peers, whose feedback patterns are associated with motivation and participation [1, 2], to conductors, whose feedback approaches correspond to singers’ emotional states and the group’s dynamics [23]. Clear and concise feedback further relates to comprehension and retention, aligning with the cognitive and emotional needs of older adults. Additionally, mutual feedback among choir members is associated with a sense of community and belonging, which is particularly relevant for older adults who may face social isolation [1]. These dynamics emphasize the importance of feedback mechanisms that are both socially sensitive and cognitively accessible.

Based on the theoretical analysis above, this study proposes the following hypotheses: **H1**: Skill-challenge balance in choral singing is positively associated with the flow of older adults. **H2**: Clear goals in choral singing are positively associated with the flow of older adults. **H3**: Immediate feedback in choral singing is positively associated with the flow of older adults.

#### Factors associated with flow (Song style and choral environment)

Song style is a factor frequently associated with flow [8, 24], yet this relationship depends heavily on contextual factors. While autonomy in musical choices correlates with individuals’ sense of agency and flow [19], this pattern is often observed differently in group settings, where collective goals and preferences must be balanced. Music featuring moderate novelty and dynamic rhythmic elements, such as variations in tempo or novel rhythmic patterns, has been observed alongside deeper immersion and heightened excitement [25]. However, overly complex or mismatched styles may correspond to disruptions in group cohesion and engagement [5, 9]. Furthermore, the alignment of song styles with participants’ skill levels and group dynamics remains underexplored, particularly in the context of older adult populations.

Likewise, the choral environment, encompassing both physical and social dimensions, exhibits important relationships with flow [22]. Optimal physical conditions, such as appropriate acoustics and lighting, are associated with enhanced immersion and fewer distractions [26]. Social dynamics, including camaraderie, mutual support, and emotional encouragement, correspond to higher engagement and reduced anxiety [1, 4, 5]. However, as Loepthien and Leipold [16] point out, flow is highly context-dependent, with elite athletes and musicians often showing different patterns of flow in different environmental setups. This suggests that while physical and social factors are important correlates, their relationship with flow may vary significantly across different populations. For some older adults, unique cognitive and social challenges, such as reduced sensory sensitivity or a heightened need for emotional connection, may correspond to different patterns in flow [17, 22].

For instance, Daffern and colleagues [23] observed that virtual environments used during the COVID-19 pandemic showed different patterns in replicating the immersive qualities compared to in-person settings. This finding underscores the importance of environmental design that aligns with the specific needs of older adults in relation to flow . Physical and social elements in choral environments appear to be important factors related to engagement, particularly for this demographic.

Building on this theoretical foundation, we propose the following hypotheses: **H4**: Choral song styles are positively associated with the flow of older adults. **H5**: The choral environment is positively associated with the flow of older adults.

### Flow, memory, and Well-being

The encoding process of memory involves transforming information into a storable form, a process that shows unique patterns when observed alongside music. Unlike emotionally matched sounds or words, music stands out in its association with positive autobiographical memories. This phenomenon relates to its simultaneous engagement of auditory perception, pattern recognition, and emotional resonance [25, 27, 28]. The relationship between music and memory encoding appears connected not only to music’s intrinsic properties, such as melody and rhythm, but also to its alignment with an individual’s emotional state and environmental context [27, 29]. For instance, music with high emotional intensity or personal relevance correlates with vivid recollections and nostalgia, corresponding to deeper and more durable memory encoding patterns [17, 30].

Well-being, a multifaceted construct encompassing life satisfaction, the presence of positive emotions, and the absence of negative emotions [31], shows positive associations with creative activities such as choral singing. These activities relate to basic psychological needs, including autonomy, competence, and relatedness [10, 32, 33]. However, the specific relationships through which flow, memory, and well-being interact in older adults remain underexplored. This gap is particularly salient within the context of choral singing, where personality traits [22, 31] and age-related cognitive changes [2, 27, 29] may correspond to unique interaction patterns.

Flow during choral singing has been found to correlate with both memory and well-being. For example, Antonini Philippe and colleagues [11] found that flow is positively correlated with life satisfaction, while Pentikäinen and colleagues [2] demonstrated that choir participation is associated with cognitive function and overall well-being in older adults. However, the assumption that flow universally correlates with cognitive processes overlooks potential variability in how older adults experience flow. For instance, some older adults may face challenges in maintaining sustained attention, which could relate to different patterns in the relationship between flow and memory [11]. Similarly, while flow is widely recognized as strongly correlated with subjective well-being [12, 14, 16], these relationships may show different patterns within the unique social and emotional dynamics of choral singing, such as social bonding and emotional resonance.

Music-related memories show a unique potential to correlate with potent emotions, nostalgia, and specific temporal associations [17, 28, 30, 31]. Engagement with musical memories shows associations with emotional well-being through comfort and positive emotions [28], corresponding to patterns of overall well-being [1, 2]. Moreover, musical memory shows correlations with therapeutic outcomes, such as reduced anxiety and improved quality of life [2], as well as flow and identity formation [8]. However, individual differences in cognitive and emotional processing [27] may correspond to different patterns in how older adults engage with musical memories. Age-related cognitive decline may further relate to different patterns in accessing and benefiting from these memories, suggesting that the relationship between musical memory and therapeutic outcomes may not be uniform across this population.

**Fig. 1:**
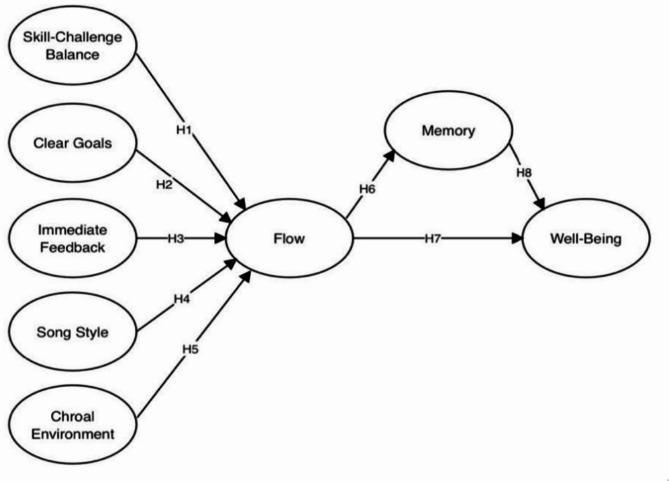
Hypothesized relationships model. (***Notes***: Memory = perceived choral memory performance)

Building on this general literature, the present study focuses on a specific, yet crucial, aspect of memory within the choral context: the participants’ own subjective perception of their memory function as it relates to their musical practice. We term this construct ‘Perceived Choral Memory Performance’. While objective cognitive tests measure memory capacity, perceived memory performance reflects the cognitive benefits that are most salient and meaningful to the participants themselves, and is thus a powerful indicator of the subjective experience of cognitive change associated with the activity. Therefore, we hypothesize the following:

**H6**: Flow during choral singing is positively associated with the perceived choral memory performance of older adults related to their choral experiences. **H7**: Flow in choral singing is positively associated with the overall well-being of older adults. **H8**: Perceived choral memory performance associated with choral singing is positively associated with the well-being of older adults. The hypothesized relationships among the variables are illustrated in Fig. 1.

## Method

### Sampling

For this study, 412 choir members were recruited from five amateur senior choirs located in Heilongjiang and Zhejiang provinces using a convenience sampling method. Participants were required to meet a predefined criterion: they must have participated in choral activities for at least six months. This non-random sampling approach was chosen due to the specific population under investigation and the geographical constraints, ensuring efficient and targeted data collection [34]. The participant pool exhibited considerable diversity, representing a wide range of socioeconomic backgrounds, including retired professionals, manual laborers, educators, and entrepreneurs. Such heterogeneity enhances the generalizability of the study’s findings to various segments of the older adult population [35]. By incorporating individuals with diverse experiences and social contexts, the study aims to provide a comprehensive understanding of the factors associated with flow, perceived choral memory performance, and well-being in senior choral settings.

The participating groups were community-based amateur choirs that typically met once or twice per week for rehearsals. The repertoire commonly included well-known Chinese folk songs and “red songs” (i.e., patriotic or revolutionary songs from the mid-20th century), which are culturally significant and familiar to this generation of older adults.

### Measurement tools

This study is grounded in Csíkszentmihályi’s flow theory, which identifies nine dimensions of flow [3]. Among these, three dimensions–skill-challenge balance, clear goals, and immediate feedback–were selected as factors related to flow. We measured skill-challenge balance with three items (e.g., “skills match challenges”), clear goals with three items (e.g., “clear role”), and immediate feedback with four items (e.g., “quick feedback,” “leader’s comments help”). This operationalization, which separates the theoretical conditions for flow (treated as predictors) from the core experiential dimensions of the state itself (used as indicators of the latent construct), allows for a direct empirical test of their relationship, consistent with our theoretical framework.

The remaining six dimensions of flow were consolidated into four, following the framework of Kawabata and Evans [36]. These dimensions were represented by single items to streamline the measurement process and reduce cognitive load for older adult participants. The four dimensions included concentration (“fully focused on singing”), immersion (“lost track of time”), sense of control (“in control of performance”), and enjoyment (“found singing enjoyable”). This streamlined design was chosen to be accessible for a diverse range of participants, including those who might find longer or more complex questions challenging.

Tailored scales were also developed to measure the choral environment and song style preferences, perceived choral memory performance, and psychological well-being. Specifically, the choral environment was assessed with three items (“comfortable space,” “supportive members,” and “sense of belonging”), and song style preferences were evaluated using four items (“familiar songs,” “variety of styles,” “uplifting melodies,” and “easy to sing”). For the primary outcomes, Perceived Choral Memory Performance were measured using a 4-item scale developed specifically for this study.

This scale drew conceptual inspiration from established instruments like the Everyday Memory Questionnaire [37], which assess subjective memory in daily life. However, to ensure high contextual relevance and face validity for our specific research question, we designed novel items tailored directly to the choral singing experience. The items included “better at lyrics,” “rarely forget plans,” “easier recall of songs,” and “quicker to memorize”.

Psychological well-being was assessed with a 4-item scale developed for this study, conceptually grounded in the multifaceted framework of well-being proposed by Diener and colleagues [38]. The items were specifically chosen to capture the nature of well-being as it pertains to this group activity by assessing affective well-being (“happier since joining,” “more energy”), social well-being (“closer to members”), and eudaimonic well-being (“life more meaningful”). This approach provides a holistic and contextually relevant measure of the subjective benefits reported by participants.

To ensure the validity of these tailored scales, they underwent a systematic development and validation process. First, an initial pool of items for each scale was generated based on established theoretical frameworks and then refined for clarity, relevance, and cultural appropriateness by a panel of six academic experts. Second, a pilot study was conducted with a small sample of the target population to assess initial reliability. The pre-test results indicated strong internal consistency for all developed constructs (Skill-challenge balance α = 0.881; Clear goals α = 0.796; Immediate feedback α = 0.847; Flow α = 0.861; Choral Environment α = 0.839; Song Style α = 0.914; Perceived Choral Memory Performance α = 0.916; Psychological Well-being α = 0.892). Finally, the robust psychometric properties of the final scales were rigorously evaluated in the main study using Confirmatory Factor Analysis (CFA), as detailed in the Measurement Model section (Sect. 4.3) and Table 1.

A 5-point Likert scale, with anchors ranging from 1 (“strongly disagree”) to 5 (“strongly agree”) and a midpoint of 3 (“neutral”), was employed for all items to ensure consistency and ease of response for participants.

### Procedure

Data were collected offline using traditional paper-and-pencil questionnaires from 412 participants. To minimize disruptions to regular choir sessions, participants were granted the flexibility to complete the questionnaire either during or after their choir meetings. This approach is grounded in its capability to produce dependable data in social science research [35, 39]. Data collection spanned a month-long period, after which all responses were digitally transcribed for subsequent analysis. To ensure the ethical integrity of the study, participant confidentiality was rigorously upheld throughout the process, and informed consent was obtained from all participants prior to data collection, in line with established ethical guidelines.

### Ethical considerations

This study was conducted in full compliance with the Declaration of Helsinki and received ethical approval from the Academic Ethics Committee of the School of Arts, Guangdong University of Petrochemical Technology. All participants provided informed consent after receiving full disclosure of the study purpose, procedures, and their rights as research participants. The investigation involved anonymous data collection through standardized questionnaires and cognitive tests, with all information stored securely and accessible only to the research team.

## Data analysis

### Common method variance (CMV) test and control

To address potential Common Method Variance (CMV), procedural and statistical controls were implemented to ensure data validity and reliability. Procedurally, respondents were assured of anonymity and voluntary participation, reducing evaluation apprehension and encouraging honest responses. A blocking strategy was also employed by interspersing unrelated personal questions between key constructs (e.g., flow, perceived choral memory performance, well-being) to disrupt response patterns, complemented by reverse-coded items to identify and mitigate inconsistent responses. Statistically, Harman’s single-factor test was conducted to assess the presence of CMV. Results indicated that a single factor accounted for 28.945% of the variance, well below the 50% threshold [40], suggesting that CMV was not a significant concern. These combined measures enhanced the validity and reliability of the study’s findings, ensuring that the data accurately reflected the constructs under investigation.

### Descriptive statistical analysis

Descriptive statistics were calculated using SPSS 26 to provide an overview of the sample characteristics and measured variables. The sample consisted of 412 participants, with 65.0% females (*n* = 268) and 35.0% males (*n* = 144). Age distribution revealed that 35.2% of participants were aged 55–59 years, 22.6% were aged 65–69 years, and 17.5% were aged 60–64 years, while those aged 70 years and above constituted 24.8% of the sample. Regarding educational background, 36.4% had completed middle school, 33.0% had completed primary school, 12.4% held a university degree, and 9.2% had a postgraduate degree or higher. In terms of occupation, the largest group was enterprise employees (26.9%), followed by workers (13.3%), civil servants (13.1%), and teachers (11.9%). Descriptive statistics for the measured variables indicated that mean scores ranged from 3.21 (G3) to 4.22 (SC1), while standard deviations ranged from 0.79 (SC1) to 1.12 (F4). Skewness and kurtosis values were within acceptable ranges, suggesting that the data were approximately normally distributed. This normality supported the use of parametric statistical methods for subsequent analyses, ensuring the robustness of the results.

### Measurement model

A rigorous evaluation of the measurement model was conducted to ensure the reliability and validity of the constructs, following the guidelines outlined by Thompson [41]for Structural Equation Modeling (SEM). Internal consistency was assessed using composite reliability (CR), with values ranging from 0.762 to 0.873 (see Table 1), all exceeding the recommended threshold of 0.70 [42]. This indicates strong reliability across all latent variables. Convergent validity was confirmed by calculating the average variance extracted (AVE), with all values surpassing the 0.50 benchmark [43], demonstrating that the constructs adequately captured the variance in their respective indicators. Additionally, all factor loadings exceeded 0.6, further supporting the model’s convergent validity [42].

Discriminant validity was assessed using the Fornell-Larcker criterion [43]. The square root of each construct’s AVE exceeded its correlations with all other constructs (see Table 1). For example, the √AVE for Well-being was 0.795, which was higher than its correlations with other constructs (ranging from 0.245 to 0.553). This pattern was consistent across all constructs, confirming that the constructs were distinct and conceptually valid.

To assess the overall fit of the measurement model, a confirmatory factor analysis (CFA) was performed using AMOS 26.0. The CFA results demonstrated an excellent model fit, with fit indices as follows: χ² = 497.666, df = 349, χ²/df = 1.426, CFI = 0.972, TLI = 0.968, IFI = 0.973, GFI = 0.926, AGFI = 0.908, RMSEA = 0.032, and Standardized RMR = 0.040. All indices met the recommended thresholds [42], indicating that the measurement model was both reliable and valid. These findings validate the constructs used in this study and demonstrate their alignment with theoretical expectations, ensuring the empirical rigor of the research.

**Table 1:** Reliability and discriminant validity analysis

Constructs	Variables	SFL	S.E.	t- value	*P*	CR	AVE	Discriminant Validity (√AVE)
**Skill-challenge Balance**	SC3	0.602				0.762	0.519	0.739
	SC2	0.756	0.111	10.593	***			
	SC1	0.789	0.108	10.648	***			
**Clear Goals**	G3	0.757				0.807	0.583	0.764
	G2	0.792	0.084	13.673	***			
	G1	0.741	0.073	13.069	***			
**Immediate Feedback**	F4	0.658				0.827	0.546	0.739
	F3	0.741	0.083	12.016	***			
	F2	0.743	0.072	12.553	***			
	F1	0.806	0.081	12.666	***			
**Song Style**	S4	0.768				0.865	0.617	0.785
	S3	0.806	0.063	16.529	***			
	S2	0.729	0.070	14.462	***			
	S1	0.834	0.060	16.802	***			
**Choral Environment**	En3	0.788				0.796	0.566	0.752
	En2	0.739	0.069	12.892	***			
	En1	0.729	0.068	13.145	***			
**Flow**	FL4	0.725				0.831	0.552	0.743
	FL3	0.699	0.076	13.116	***			
	FL2	0.804	0.078	14.038	***			
	FL1	0.740	0.076	13.913	***			
**Perceived Choral Memory Performance**	M4	0.749				0.854	0.596	0.772
	M3	0.670	0.062	12.9	***			
	M2	0.852	0.064	16.241	***			
	M1	0.805	0.064	15.95	***			
**Well-being**	W4	0.807				0.873	0.633	0.795
	W3	0.825	0.055	18.428	***			
	W2	0.743	0.061	15.569	***			
	W1	0.804	0.056	17.313	***			

Notes: SFL = Standardized Factor Loading; S.E.=Standard Error; t value shows the significance of the factor loadings; *P* denotes the significance level (*** indicates *p* < .001); CR = Composite Reliability; AVE = Average Variance Extracted; HTMT = Heterotrait-Monotrait Ratio

**Table 2:** Independent samples t-Test results for flow and Well-being by gender

Variable	Gender	Mean	Std. Deviation	Equal Variances Assumed	t	df	*p*	95% CI
**Flow Mean**	Male	3.564	0.745	Yes	-2.66	410	0.008	− 0.37, − 0.06
	Female	3.777	0.788					
**Well-Being Mean**	Male	3.208	0.803	Yes	-6.99	410	< 0.001	− 0.73, − 0.41
	Female	3.780	0.786					

### Independent samples t-Test

An independent samples t-test was conducted to examine gender differences in well-being and flow. The results indicated that females reported significantly higher well-being levels (M = 3.78, SD = 0.79) compared to males (M = 3.21, SD = 0.80), t (410) = -6.99, *p* < .001, 95% CI [-0.73, − 0.41], with a large effect size (Cohen’s d = 0.72) [44] (see Table 2). These findings indicate that gender differences in well-being are both statistically significant and practically meaningful. For flow, females also reported slightly higher levels (M = 3.78, SD = 0.79) than males (M = 3.56, SD = 0.75), t (410) = -2.66, *p* = .008, 95% CI [-0.37, − 0.06], with a small to moderate effect size (Cohen’s d = 0.28) [44]. While this difference is statistically significant, its practical significance appears limited due to the smaller effect size. Levene’s test confirmed equal variances for both well-being (F = 0.077, *p* = .781) and flow (F = 0.846, *p* = .358), validating the assumptions of the t-test. Overall, these results highlight meaningful gender differences in well-being and a smaller, though statistically significant, difference in flow.

### Structural equation modeling and hypotheses examination

#### Structural model

To explore the multivariate relationships among latent variables, Structural Equation Modeling (SEM) was employed as the primary analytical framework. SEM is particularly suitable for this study due to its ability to simultaneously evaluate multiple relationships among variables, including potential mediating relationships—such as the relationship of perceived choral memory performance with flow and well-being—while assessing the overall fit of the proposed model [45]. This integrated approach facilitates a nuanced understanding of the complex relationships among the variables under investigation.

**Fig. 2:**
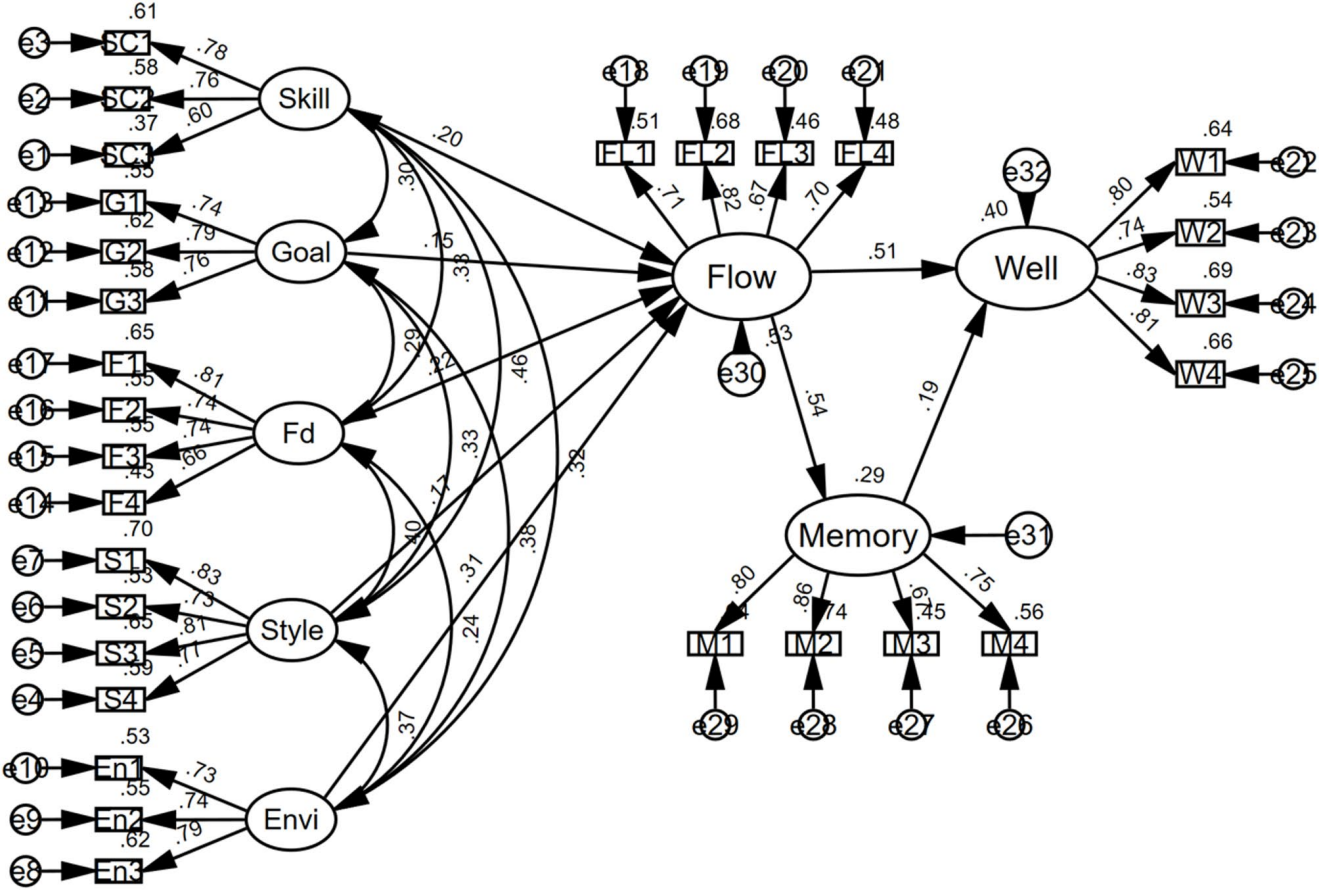
Final Structural Equation Model with Standardized Path Coefficients. ***Note***: The ‘e’ variables (e.g., e1 to e29) in small circles represent the residual error terms for each corresponding observed variable (the squares); Skill = Skill–challenge balance; Fd = Immediate feedback; style = Song style; Envi = Choral Environment; Well = Well-Being; Memory = perceived choral memory performance

The structural model demonstrated a strong alignment with the empirical data, as evidenced by the following fit indices: χ² = 581.739, df = 359, χ²/df = 1.620, CFI = 0.959, TLI = 0.953, IFI = 0.959, and RMSEA = 0.039. These indices meet the established benchmarks for good model fit [42], indicating that the proposed model accurately represents the observed data. The parameter estimates obtained through maximum likelihood estimation are visually presented in Fig. 2 (Estimates of Structural Equation Modeling). Additionally, the results of the indirect relationships analysis, conducted using bootstrap procedures, are summarized in Table 4 (Standardized Indirect Effects with Bootstrap Confidence Intervals). These results indicate the significance and stability of the indirect relationships, offering support for the theoretical framework.

#### Hypotheses testing

The analysis of hypotheses H1 through H5 examined the relationships between the five factors and “flow.” The results revealed significant positive associations between all five factors and flow. Specifically, skill-challenge balance (β = 0.196, t = 3.300, *p* < .001), clear goals (β = 0.148, t = 2.643, *p* = .008), immediate feedback (β = 0.218, t = 3.875, *p* < .001), song style (β = 0.174, t = 2.952, *p* = .003), and choral environment (β = 0.312, t = 5.233, *p* < .001) all showed significant positive associations with “flow” (see Table 3). Among these factors, choral environment demonstrated the strongest association with “flow,” as indicated by the highest standardized path coefficient (β = 0.312).

Expanding the analytical scope, hypotheses H6 and H7 examined the relationships between “flow” and both perceived choral memory performance and well-being. The results indicated significant positive associations between “flow” and perceived choral memory performance (β = 0.539, t = 8.398, *p* < .001) and between “flow” and overall well-being (β = 0.507, t = 7.149, *p* < .001) (see Table 3). Hypothesis H8 tested the relationship between perceived choral memory performance and well-being, which was also found to be significant (β = 0.190, t = 3.112, *p* = .002) (see Table 3). These findings indicate significant positive associations among “flow,” perceived choral memory performance, and well-being in the context of older adult choral participation.

**Table 3:** Path coefficients of the structural model

Path	Std.	Unstd.	S.E.	t-value	*p*-value
H1: Skill → flow H2: Clear Goals → flow H3: Immediate feedback → flow H4: Song style → flow H5: Choral Environment → flow H6: flow →Memory H7: flow → Well-Being H8: Memory→ Well-Being	0.196	0.221	0.067	3.300	***
	0.148	0.145	0.055	2.643	0.008
	0.218	0.206	0.053	3.875	***
	0.174	0.150	0.051	2.952	0.003
	0.312	0.305	0.058	5.233	***
	0.539	0.607	0.072	8.398	***
	0.507	0.558	0.078	7.149	***
	0.190	0.185	0.060	3.112	0.002

*Notes*: Std.=Standardized Coefficient; Unstd.=Unstandardized Coefficient; S.E.=Standard Error; ***p < .001; Skill = Skill–challenge balance; Memory = perceived choral memory performance

### Indirect relationship analysis

To examine the indirect relationships between the five factors–skill-challenge balance, clear goals, immediate feedback, song style, and choral environment–and perceived choral memory performance and well-being through “flow,” the bias-corrected bootstrap method with 5000 resamples was employed [45]. This method provides reliable confidence intervals for indirect relationships, thereby enhancing the robustness of the analysis.

The results indicated significant indirect relationships between all five factors and perceived choral memory performance through “flow.” Specifically, the standardized indirect coefficients were as follows: skill-challenge balance (0.106, 95% CI [0.055, 0.220]), clear goals (0.080, 95% CI [0.033, 0.148]), immediate feedback (0.118, 95% CI [0.070, 0.193]), song style (0.094, 95% CI [0.033, 0.157]), and choral environment (0.169, 95% CI [0.122, 0.260]). Among these, choral environment demonstrated the strongest association. Similarly, significant indirect relationships with well-being through “flow” were observed: skill-challenge balance (0.120, 95% CI [0.060, 0.247]), clear goals (0.090, 95% CI [0.036, 0.161]), immediate feedback (0.133, 95% CI [0.077, 0.211]), song style (0.106, 95% CI [0.036, 0.174]), and choral environment (0.190, 95% CI [0.135, 0.281]). Once again, choral environment exhibited the strongest association.

In addition to these findings, the analysis revealed a significant indirect relationship between perceived choral memory performance and well-being through “flow” (0.102, 95% CI [0.054, 0.179]), highlighting the association patterns among these variables. These results further emphasize the statistical significance of “flow” as an intervening variable in these relationships. Detailed results are presented in Table 4.

**Table 4:** Standardized indirect relationships with bootstrap confidence intervals

Path	Point estimate	Bootstrap 5000 times		
		Bias-Corrected		
		95% CI		
		SE	Lower	Upper
Skill → Memory	0.106	0.049	0.055	0.220
Clear Goals → Memory	0.080	0.035	0.033	0.148
Immediate Feedback → Memory	0.118	0.038	0.070	0.193
Song style→ Memory	0.094	0.038	0.033	0.157
Choral Environment → Memory	0.169	0.042	0.122	0.260
Skill →Well-being	0.120	0.056	0.060	0.247
Clear Goals → Well-being	0.090	0.039	0.036	0.161
Immediate Feedback → Well-being	0.133	0.041	0.077	0.211
Song style → Well-being	0.106	0.042	0.036	0.174
Choral Environment → Well-being	0.190	0.045	0.135	0.281
Flow →Well-being	0.102	0.038	0.054	0.179

***Notes***: Point estimate = Standardized Estimates; SE = Indirect Relationships-Stand Error; Skill = Skill–challenge balance; Memory = perceived choral memory performance

## Discussion

This study explores the factors associated with flow among older adults in choral settings, particularly their connections to perceived choral memory performance and well-being. As a general observation, participants reported largely positive experiences, with mean scores for most measured variables falling in the mid-to-positive range on the 5-point scales. This suggests that, overall, choral singing is a highly engaging and beneficial activity for this demographic. Delving deeper, our results indicate that skill-challenge balance, clear goals, and immediate feedback are positively associated with flow, consistent with Csíkszentmihályi’s theory [3] and supporting Hypotheses 1–3. This reaffirms the theory’s relevance to collective musical engagement among older adults, a domain less studied. However, unlike Kang [5], who identified skill as showing the strongest relationship with flow among professional music teachers, or Ding and Hung [4], who emphasized skill performance in music festival visitors, our findings highlight the choral environment as showing the strongest association (H5), with immediate feedback showing the second strongest relationship. While Ding and Hung [4] acknowledged the role of atmosphere, our results suggest that the choral environment–combining physical comfort and social dynamics–shows particularly strong associations with flow in this context. This pattern may reflect the collective nature of choral singing and the diverse skill levels of older adults, where factors like peer encouragement and conductor guidance correlate with focus and cognitive outcomes, showing stronger associations than technical mastery [2, 20, 21].

Song style also shows significant associations with flow, as evidenced by Hypothesis 4, complementing the choral environment’s relationship. The notable correlation with “red songs”--Chinese revolutionary songs–extends beyond emotional resonance [8, 24]. Rooted in China’s collectivist values and historical narratives, these songs correspond with autobiographical memories and emotional depth [30, 31]. By connecting personal memories to a collective legacy, they appear to relate to focus and enjoyment, corresponding with immersion in this cultural setting [17, 31]. This highlights how song selection correlates with collective nostalgia, suggesting potential applications for community programs [25]. However, a more nuanced perspective on nostalgia is warranted. Our interpretation simplifies nostalgia as a uniformly positive experience, yet research indicates it is a complex, double-edged emotion. For individuals inclined to rumination, nostalgia can amplify negative affect [46]. The psychological benefits of nostalgia are often tied to a reflective, rather than a brooding, cognitive style when recalling the past [47]. Our cross-sectional design did not assess these individual differences in personality or coping styles, and exploring how these factors moderate the affective outcomes of nostalgia in collective singing contexts presents a rich and important avenue for future research.

The study also highlights strong positive relationships among flow, perceived choral memory performance, and well-being, supporting Hypotheses 6, 7, and 8. Flow shows a positive association with perceived choral memory performance (H6), consistent with Pentikäinen and colleagues [2], who found that choir singing correlates with improved encoding through sustained engagement. It also shows a direct positive relationship with well-being (H7), aligning with Ding and Hung [4], who found associations between flow in musical contexts and emotional satisfaction. Furthermore, perceived choral memory performance shows a positive relationship with well-being (H8), as observed by Janata and colleagues [30], where music-evoked recall corresponded with mental health indicators.

All five factors–skill-challenge balance, clear goals, immediate feedback, choral environment, and song style–show significant indirect relationships with perceived choral memory performance and well-being through flow, with the choral environment showing the strongest indirect relationship. This underscores its position as showing the strongest association with flow (H5), where social support and real-time reinforcement correspond with cognitive and emotional outcomes [1, 2, 20, 21]. Additionally, the analysis revealed an indirect relationship between flow and well-being through perceived choral memory performance, an emergent finding not initially hypothesized. Flow shows associations with perceived choral memory performance during choral activities, with music’s rhythm and emotions potentially serving as mnemonic anchors [30]. For older adults, “red songs” may strengthen this relationship through culturally rooted recall [25, 28], corresponding with well-being through emotional arousal and identity preservation amid cognitive decline [1, 2].

A notable gender difference also emerged, with women reporting greater well-being associated with choral participation than men. This aligns with Anglim and colleagues [32], who linked well-being to personality and social dynamics. Gender role theory offers additional insight, suggesting that women, often socialized to prioritize relationships, may derive more satisfaction from the camaraderie and emotional support of choral singing [1, 2]. Habe and colleagues [12] similarly observed higher well-being scores among women in collective musical activities, potentially related to social-emotional engagement patterns. By contrast, men, shaped by norms emphasizing independence, may show different patterns of connection with these relational aspects. This difference, as Moral-Bofill and colleagues [22] noted, may reflect varying emotional needs in older adults. Tailored approaches, such as discussion groups for women to strengthen bonds or repertoires appealing to men’s emotional preferences, could be considered for diverse participants [1, 32].

## Limitations and future directions

First, the cross-sectional design of this study limits our ability to establish causal relationships between the variables. While our findings reveal significant associations between factors like the choral environment, flow, perceived choral memory performance, and well-being, we cannot infer causality. Future research would benefit from longitudinal designs or experimental methods to explore the directionality and potential causal mechanisms underlying these relationships more definitively.

Second, our study utilized a convenience sampling method, with participants recruited from specific provinces in China. While this approach was practical for accessing our target population, it may limit the generalizability of our findings to older adults in different cultural contexts or geographical regions. Additionally, the sample size for male participants (*n* = 144), while substantial, may not have sufficient statistical power for a stable multi-group SEM analysis to robustly test for gender differences in the model’s path coefficients. Future studies with larger, more diverse, and gender-balanced samples are needed to test the robustness of these associations across different populations.

Finally, this study relied on self-report measures, including several scales adapted specifically for the choral context, such as the Perceived Choral Memory Performance scale. While these scales demonstrated strong psychometric properties within our sample and offered high contextual validity, they are not standardized instruments. We acknowledge that self-reported data can be subject to biases, such as social desirability. Future research could corroborate our findings by incorporating objective measures (e.g., standardized cognitive tests for memory), observational data, or qualitative methods that capture participants’ direct comments and experiences, to provide a more comprehensive, multi-method understanding of the choral singing experience.

## Conclusion and implications

This study illuminates the intricate relationships among flow , perceived choral memory performance, and well-being in older adults participating in choral activities. Our findings reveal significant associations between several factors (skill-challenge balance, clear goals, immediate feedback, song style, and choral environment) and flow, which in turn show robust relationships with perceived choral memory performance and well-being. A notable pattern in our results is the significant indirect relationship between flow and well-being through perceived choral memory performance, with the choral environment demonstrating the strongest association with flow among all factors examined. These results not only align with Csíkszentmihályi’s flow theory [3] but extend our understanding to valuable contexts involving group dynamics and aging populations, which have been underrepresented in prior research [11, 13].

This study offers several important contributions. Theoretically, it extends flow theory into the under-researched domain of collective musical engagement among older adults. By empirically demonstrating that the choral environment can be a more powerful antecedent of flow than individual factors in this context, our findings enrich the theory by highlighting the profound role of social and environmental dynamics. Furthermore, we establish a novel pathway linking flow, context-specific cognitive appraisal (Perceived Choral Memory Performance), and multifaceted well-being, thus expanding the explanatory power of the theory in aging populations.

Practically, these findings provide actionable insights for multiple stakeholders. For choir directors and community organizers, our results underscore the critical need to cultivate a supportive and positive environment. Beyond focusing on technical skill, leaders should prioritize creating socially inclusive spaces and consider culturally resonant repertoires, such as “red songs.” The significant connection found in our study suggests there is considerable value in incorporating nostalgic elements in repertoire selection to enhance engagement [25, 30]. For health professionals and policymakers, this study provides robust evidence that community choral programs are a promising, low-cost strategy. They represent an evidence-informed activity that positively correlates with the psychological well-being and subjective cognitive vitality of the aging population, a finding consistent with a growing body of literature [2, 17].

While our cross-sectional design examines associations rather than causal relationships, the strength and consistency of these relationships provide valuable insights into the complex interplay of psychological factors in older adult choral contexts. Beyond this methodological consideration, the study’s focus on a Chinese sample and specific song styles, such as “red songs,” presents opportunities for cross-cultural examination. Different cultural and musical contexts may reveal additional patterns, suggesting rich avenues for further exploration.

Future research would benefit from longitudinal designs or experimental methods to further explore the directionality and mechanisms of these relationships. Such approaches would complement our findings by examining potential causal connections between flow, perceived choral memory performance, and well-being in choral contexts. Additionally, investigating diverse cultural contexts and repertoires would test the robustness of these associations across different populations. Beyond music, examining relationships between other art forms and well-being variables, as well as how institutional structures and social support correspond with participation in creative activities, could expand our understanding of psychological health correlates. These research directions would build upon the significant foundation established in this study regarding arts participation and mental well-being across diverse populations.

## Supplementary Information

Below is the link to the electronic supplementary material.

Supplementary Material 1

## Data Availability

The datasets generated and analyzed during this study are available from the corresponding author upon reasonable request.
