# Supplementary material for: Flow in choral singing: associations with perceived choral memory performance and well-being among older adults
Source: BMC Psychol. 2025 Aug 11;13:892. doi: 10.1186/s40359-025-03276-w (PMC12341315; doi:10.1186/s40359-025-03276-w)
Supplement: Supplementary file 1 — Supplementary Material 1 [file 40359_2025_3276_MOESM1_ESM.docx]

**Ethics Statement**

This study was conducted in accordance with the ethical principles of the Declaration of Helsinki and relevant ethical guidelines for psychological research in China. The research involved only the collection of basic sociodemographic information without any personally identifiable information from participants, thus protecting their anonymity.

All participants provided informed consent before participating in the study. They were fully informed about the purpose of the research, the voluntary nature of their participation, their right to withdraw at any time without consequences, and how their data would be used and protected.

This research did not involve any invasive procedures, medical interventions, or experiments that could potentially cause physical or psychological harm to participants. The study consisted only of questionnaire surveys for data collection and cognitive tests administered to elderly participants.

Data were collected anonymously, with sociodemographic information gathered only for analytical purposes. All research data were stored securely with password protection and accessible only to the research team. Upon completion of their participation, participants were debriefed about the study's objectives and provided with contact information should they have any questions or concerns.
